# Two case reports: Whole genome sequencing of two clinical macrolide-resistant *Mycoplasma pneumoniae* isolates with different responses to azithromycin

**DOI:** 10.1097/MD.0000000000004963

**Published:** 2016-09-23

**Authors:** Shaoli Li, Hongmei Sun, Fei Liu, Yanling Feng, Hanqing Zhao, Guanhua Xue, Chao Yan

**Affiliations:** a Department of Bacteriology, Capital Institute of Pediatrics; b Microbial Genome Research Center, CAS Key Lab of Pathogenic Microbiology and Immunology, Institute of Microbiology, Chinese Academy of Sciences, Beijing, China.

**Keywords:** adhesion P1, erythrocyte sedimentation rate, macrolide-resistant *Mycoplasma pneumoniae*, single nucleotide polymorphisms, whole genome sequencing

## Abstract

**Background:**

Cases of macrolide-resistant *Mycoplasma pneumoniae* have increased rapidly since 2000, especially in Asia. Patients infected with macrolide-resistant *M pneumoniae* usually present with severe *M pneumoniae* pneumonia. The aim of this study was to identify indicators for whether children at an early stage of *M pneumoniae* infection develop mild or severe pneumonia.

**Case Summary:**

Herein, we retrospectively reviewed 2 pediatric cases caused by macrolide-resistant *M pneumoniae*, but with markedly different severity of pneumonia. First, we compared the clinical courses of the patients, then isolated the pathogens and tested their response to macrolides, then finally, carried out whole genome sequencing of these isolates. Despite the difference in clinical presentation of the infection, both isolates exhibited a high level of resistance to macrolide antibiotics. Analysis of clinical data showed that the erythrocyte sedimentation rate in blood samples of the patients in the early stages of disease varied greatly. Genome sequence analysis revealed single nucleotide polymorphisms mainly focused on adhesin P1, which is involved in the pathogenicity of *M pneumoniae*.

**Conclusion:**

The differences of erythrocyte sedimentation rate in the early stage of *M pneumoniae* pneumonia and mutations in P1 protein may help us to distinguish between severe or mild disease after infection with macrolide-resistant *M pneumoniae*. These findings could lead to the development of screening assays that will allow us to distinguish severe or mild *M pneumoniae* pneumonia early.

## 1 Introduction

*Mycoplasma (M) pneumoniae* is a major cause of community-acquired respiratory tract infections, especially in children and young adults, and its positive rates range from 10% to 30%, and may reach 50% to 80% in years of peak incidence or during local outbreaks.^[1,2]^ It is transmitted through aerosols, and the severity of infection varies from mild upper respiratory tract infection to severe pneumonia.^[3]^*M pneumoniae* infections can be treated with macrolides, which are generally considered to be the first-choice antibiotics for children; tetracycline and fluoroquinolones are generally not recommended for use in children because of potential adverse effects.^[4,5]^ However, the prevalence of macrolide-resistant *M pneumoniae* strains has increased rapidly since 2000, especially in Asian countries (In China, in 2012, 100% of *M pneumoniae* isolates were macrolide resistant; Japan, 100% in 2015; Korea, 64.5% in 2012).^[5–13]^ Point mutations in the 23S ribosomal RNA gene are known to be the major mutations that give rise to drug resistance in *M pneumoniae*.^[5]^ Patients who have pathogens with point mutations in the 23S ribosomal RNA gene are usually defined as macrolide-resistant patients.

Herein, we retrospectively reviewed 2 cases caused by macrolide-resistant *M pneumoniae* in pediatric patients who both had A2063G mutations in the 23S rRNA gene, but have different degrees of severity of *M pneumoniae* pneumonia. We compared the clinical courses of disease, and then carried out whole genome sequencing of these isolates.

## 2 Case reports and methods

### 2.1 Ethics statement

The present project was performed in compliance with the Helsinki Declaration (Ethical Principles for Medical Research Involving Human Subjects) and was approved by the research board of the Ethics Committee of the Capital Institute of Pediatrics, Beijing, China. All patient data were anonymously reported.

### 2.2 Case 1

A 6-year-old girl with cough and sputum for the prior 9 days and a fever for the prior 8 days visited our hospital. Prior to admission to our hospital, blood counts analyzed on 3 different days in another hospital were normal (white blood cell [WBC] count, percent neutrophils, and percent lymphocytes) and C-reactive protein (CRP) level was continuously raised (9, 21, 44 mg/L; NR <8 mg/L). The patient was diagnosed with atypical pneumonia empirically and initially treated with azithromycin (an unremarkable medical history), but her clinical conditions had not improved.

For further evaluation and treatment she was transferred to our hospital. On day 5 after admission to our hospital, she still had persistent fever and cough, with fever of up to 39.8°C twice a day, rash and sputum. Coarse crackles were heard in her right upper thorax, and a spot was visible on her right lung by chest radiograph. The titer of anti-mycoplasma IgM antibody was 1:640. Based on the clinical symptoms and further laboratory tests (CRP 41.6 mg/L, erythrocyte sedimentation rate (ESR) 62 mm/60 min) (Table 1), pediatricians diagnosed severe *M pneumoniae* pneumonia. Intravenous azithromycin (10 mg/kg/d) was restarted, but without clinical improvement during the next 5 days, and imipenem was added on day 2 after admission because of the increasing CRP. The results of microbiological tests of the samples obtained at admission were negative for respiratory viruses (respiratory syncytial virus, adenovirus, influenza viruses, parainfluenza viruses, human metapneumovirus, human bocavirus, coronaviruses) and some (atypical) bacteria (*Streptococcus pneumoniae, Haemophilus influenzae, Staphylococcus aureus, Mycobacterium tuberculosis, Chlamydophila pneumoniae,* and *Legionella pneumophila*). On day 6 after admission, her anti-EBV IgM and IgG were positive, the culture of *M pneumoniae* also was positive. Ganciclovir was thus added, in addition to the other treatment.

**Table 1:**
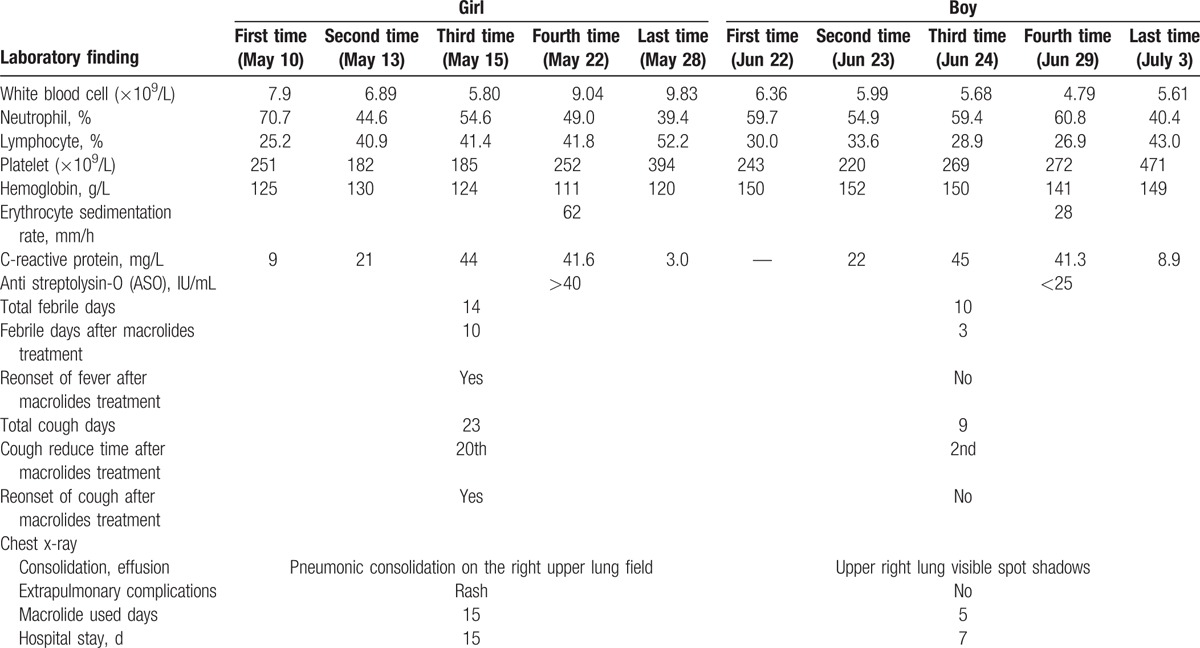
Clinical course, laboratory, and radiologic findings in 2 children with macrolide resistant *M pneumoniae* infection.

On day 10 after admission in our hospital, the patient's temperature stabilized, but she had a worsening dry cough. The pediatrician suggested continuing the treatment. On day 12 after admission, her clinical symptoms and chest radiography findings had markedly improved, with the level of CRP reduced to 3.0 mg/L, and other indicators normal. On day 14 after admission in our hospital, she was given a third round of oral azithromycin replacing the intravenous treatment. During her illness, this patient had fever for a total of 14 days, with recurrent febrile days even after macrolide treatment for 10 days. She coughed a total of 23 days, and cough relief after macrolide treatment was on the 12th day of treatment in our hospital. A marked improvement was observed on the 15th day of hospitalization, and she was discharged.

### 2.3 Case 2

A 12-year-old boy, who was otherwise previously healthy, was admitted to our hospital after suffering for 6 days from a dry cough with fever up to 38.8°C. Prior to admission to our hospital, blood samples were analyzed on 3 different days at another hospital, revealing normal WBC counts and increasing CRP (12, 22, 45 mg/L; NR <8 mg/L), ESR 28 mm/60 min. His breath sounds were normal, and no abnormal physical findings were observed. The chest radiograph showed pneumonic consolidation on the right upper lung field. Thus, he was diagnosed with atypical pneumonia. After taking a pharyngeal swab sample and serum, he was initially treated with intravenous azithromycin 10 mg/kg/d for 5 days based on the pediatrician's empirical judgment. After 3 days in our hospital, his temperature was falling and his cough improving. After 5 days of antibiotic therapy here, no relapse (fever and cough) was observed, and his chest radiograph also showed improvement. This case was diagnosed as mild *M pneumoniae* pneumonia because anti-mycoplasma IgM antibody was present at a titer of 1:160. Further microbiological tests of blood, nasopharyngeal swab, and sputum samples continued to show positive *M pneumoniae* DNA and no other pathogen. He was discharged quickly.

## 3 Methods

### 3.1 Culture of *M pneumoniae* isolates

*M pneumoniae* isolates were obtained by cultivation of specimens in pleuropneumonia-like organism medium (PPLO broth [Becton, Dickinson and company, Sparks, MD, USA], yeast extract [10%, Oxoio LTD, England], unheated horse serum [20%, Lanzhou National Hyclone Bio-Engineering Co LTD, Lanzhou, China], glucose [50%, CR Double-Crane Pharmaceuticals Co, Ltd, China], phenol red [0.4%, Amresco, Solon, OH], and penicillin [1000 U/mL, North China pharmaceutical Group Corporation, China]) at 37°C in a BSL-2 laboratory for several days until the broth had color changes. Each sample was continuously passaged 4 to 8 times.

*M pneumoniae* DNA was extracted using the QIAamp Mini DNA kit (Qiagen, Hilden, Germany) according to the manufacturer's instructions. They were then tested by PCR for the detection of macrolide resistance genes in 23S rRNA and P1, and by Multiple Locus Variable Number of Tandem Repeats (VNTR) Analysis (MLVA) typing using our previous methods.^[14,15]^

### 3.2 Minimal inhibitory concentration testing (MIC)

Antimicrobial susceptibility testing was carried out using a broth microdilution method from the Clinical and Laboratory Standards Institute (CLSI document M43A). Erythromycin, azithromycin, medemycin, tetracycline, and fluoroquinolones were used in this analysis; *M pneumoniae* reference strain FH (ATCC 15531) was used as a drug-susceptible control strain. All tests were performed in triplicate.

### 3.3 Genomic DNA preparation, library construction, and DNA sequencing

*M pneumoniae* genomic DNA was extracted using the QIAamp Mini DNA kit (Qiagen, Hilden, Germany) according to the manufacturer's instructions. The DNA was sonicated using a Diagenode Bioruptor (Diagenode SA, Liège, Bele Mauve). The Illumina Hiseq2000 sequencing platform was used in this study. SOAPsnp was used to score single nucleotide polymorphisms (SNPs) from aligned reads.^[16]^ The short reads were first aligned onto the M129 reference genome using the SOAP2 program.^[17]^ To obtain reliable alignment hits, a maximum of 2 mismatches was allowed between the read and the reference sequence. For paired-end data, mapping locations for each read were restricted to sites within 500 bp of the mapping location of the partner sequence. For the other data, strain-specific SNPs were manually reviewed by taking into account whether SNPs were detected by MAUVE. We used BRIG (BLAST Ring Image Generator) to show the similarity between a reference sequence M129 and the 2 isolate sequences as concentric rings.^[18]^ Finally, we designed PCR primers for assays to join the scaffolds using an ABI-3730 genetic analyzer (Applied Biosystems, Foster City, CA).

## 4 Results

### 4.1 Sequence analysis of 23S rRNA gene and P1 and MLVA typing

The 2 patients’ *M pneumoniae* isolates harbored A2063G mutations in domain V of the 23S rRNA gene but no other commonly reported mutations. They belonged to type 1 according to traditional P1 typing, and the MLVA type of Case 1 was type 5-3-5-6-2, and Case 2 was 6-3-5-6-2.

### 4.2 MICs of clinical isolates

Table 2 shows the minimal inhibitory concentrations of 5 drugs on the isolates from the 2 patients.

**Table 2:**
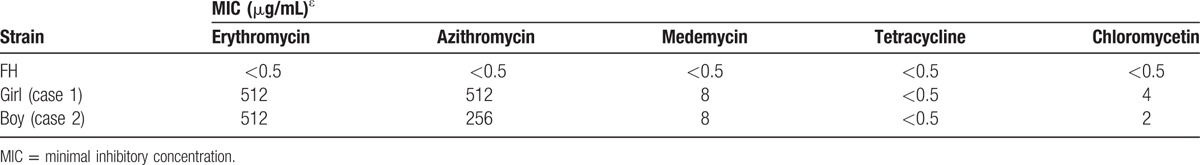
Minimal inhibitory concentrations of 2 clinical *M pneumoniae* isolates.

### 4.3 Genome sequences of 2 clinical isolates

**Figure 1:**
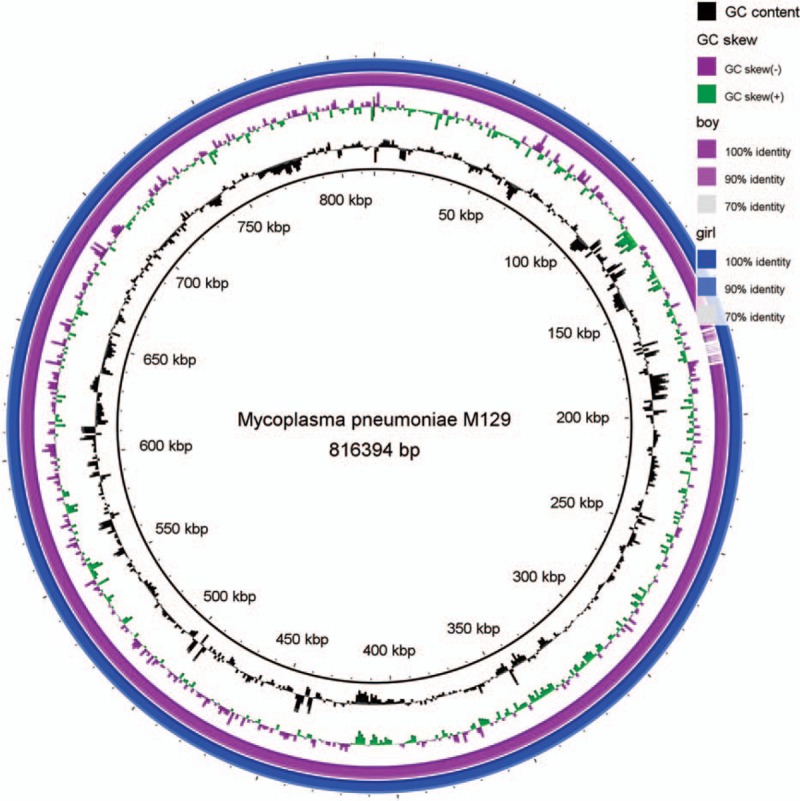
A circular plot of genome diversity between the clinical isolates and the reference genome M129, drawn with BLAST Ring Image Generator (BRIG).

Genome sequence analysis revealed that the whole genome sequences of the 2 clinical *M pneumoniae* strains were 816,498 bp (Case 1) and 801,203 bp (Case 2) in length; they had a G+C content of 39%. Comparative genomic analyses were performed using the genome sequence of M129 as a reference. Figure 1 shows a circular plot of genome diversity between the 2 clinical isolates and the reference genome M129, drawn with BRIG. A small difference was seen at around 180 kbp. SNP analysis showed that the differences between isolates were mainly observed in a hypothetical protein and adhesin P1 (or cytadherence protein) (Table 3).

**Table 3:**
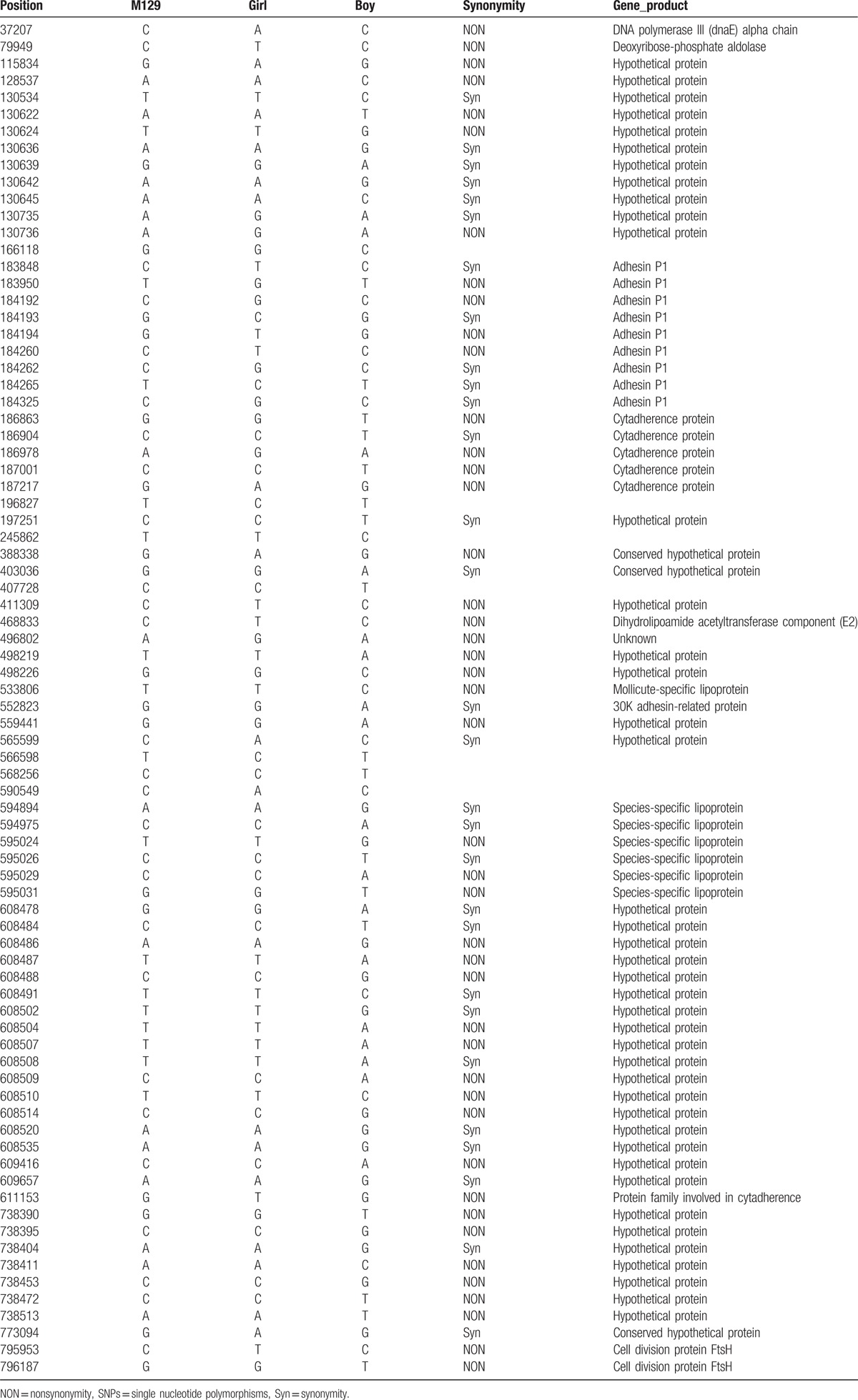
SNPs of 2 *M pneumoniae* isolates according to reference strain M129.

## 5 Discussion

Macrolides are generally considered to be the first-choice agents for the treatment of *M pneumoniae* infection.^[1–4]^ In 2000, *M pneumoniae* showing resistance to macrolides was isolated from clinical samples obtained from Japanese pediatric patients with community-acquired respiratory tract infections. Since then, the prevalence of macrolide-resistant *M pneumoniae* isolates in pediatric patients has increased rapidly.^[5–13]^

*M pneumoniae* macrolide resistance has been associated with point mutations in domain V of the 23S rRNA gene of *M pneumonia*, and usually causes severe pneumonia.^[5]^ However, in our study, the 2 isolates were both macrolide-resistant *M pneumoniae*, based on the presence of an A-to-G transition at position 2063 of the 23s rRNA gene. However, the girl, Case 1, had severe *M pneumoniae* pneumonia with longer-lasting fever and cough, which only improved after the second course of azithromycin. In contrast, the boy, Case 2, had mild *M pneumoniae* pneumonia, which only required 3 days of macrolide treatment. In previous studies, severe *M pneumoniae* pneumonia patients have been shown to exhibit higher CRP (CRP >40 mg/L) and ESR (ESR >30 mm/h) than normal, and these parameters were considered a useful marker for predicting the efficacy of macrolides and helping clinicians make better clinical decisions in children with macrolide-resistant *M pneumoniae* infection.^[19]^ In this study, Case 1 had severe *M pneumoniae* pneumonia with an ESR of 62 mm/h, whereas Case 2 had mild *M pneumoniae* pneumonia with an ESR of 28 mm/h. This result was consistent with previous reports: the value of ESR may be helpful for early identification of severe *M pneumoniae* pneumonia in children. In contrast, they had the same trend of change in CRP, which was different from previous studies.^[19,20]^

Investigation of antibiotic resistance showed that both isolates had a high level of resistance to erythromycin and azithromycin and low level of resistance to medemycin; but they were susceptible to tetracycline and chloromycetin. Though we demonstrated in vitro susceptibility of macrolide-resistant *M pneumoniae* to tetracycline and chloromycetin, neither of them are recommended for use in children, because tetracyclines can cause permanent dental discoloration and fluoroquinolones have potential for damaging cartilage.^[3,4]^

There are several possible explanations for the observation that isolates from patients were resistant to macrolide antibiotics, even though the patients had markedly different courses of disease. First, although resistant to macrolide antibiotics, the MIC value for azithromycin of the isolate from Case 2, with milder disease, was lower than that of Case 1, with more severe disease. Second, the inflammatory response to *M pneumoniae* infection may play a crucial role in the pathogenesis of clinical disease; the girl, Case 1, with more severe disease, had previously (4 years prior) developed asthma, from which she fully recovered; but in the year preceding this infection she had recurrent respiratory tract infections, suggesting that her immune system may be somewhat depressed. Third, Case 1 also had a mixed infection with Epstein–Barr virus, which may have increased the severity of her illness. Fourth, although isolates from both patients belonged to type 1 according to traditional P1 typing, the MLVA type of Case 1 was type 5-3-5-6-2, and Case 2 was 6-3-5-6-2, differing only at the *Mpn1* locus. It is not known whether variability at the *Mpn1* locus is associated with severity of disease and no conclusion can be reached based on the number of cases in the present study. Last, but most importantly, whole genome sequence analysis showed that the main differences between the 2 isolates were in hypothetical proteins and adhesin P1. The P1 protein plays an important role in the attachment to human respiratory epithelial cells, and is involved in pathogenicity of *M pneumoniae*, so mutations in this protein may change the virulence of *M pneumoniae* strains.^[21–24]^ We speculate that identification of mutations in the P1 protein could aid in distinguishing severe from mild disease after infection with macrolide-resistant *M pneumoniae*. As the function of the hypothetical proteins is unknown, we cannot rule out that mutations in these hypothetical proteins could also affect disease severity.

In conclusion, ESR in the early stage of *M pneumoniae* pneumonia and mutations in P1 protein may be effective indicators for distinguishing severe or mild disease, and in vitro resistance to macrolides does not necessarily equate to clinical failure. In a healthy child with a macrolide-resistant *M pneumoniae* infection, azithromycin still showed a favorable clinical and microbiological response. However, a patient with related primary diseases, like asthma or immunodeficiency disease, may have severe pneumonia caused by macrolide-resistant *M pneumoniae*, azithromycin may have a poor effect and need several rounds of treatment, or other avenues of treatment may need to be explored. Knowledge of the health history of patients is thus very important for the ability to predict the severity of the disease at the early stages, and early diagnosis of severe macrolide-resistant *M pneumoniae* is crucial for the best management of these patients.

Our study, however, had several limitations. There were only 2 patients in this study. We have not demonstrated the relationship between the severity of disease and the presence of mutations. Further prospective and large-scale studies are needed to resolve this uncertainty.
